# Expression of Estrogen Receptor α by Decidual Macrophages in Preeclampsia

**DOI:** 10.3390/biomedicines9020191

**Published:** 2021-02-14

**Authors:** Polina Vishnyakova, Anastasiya Poltavets, Maria Nikitina, Konstantin Midiber, Liudmila Mikhaleva, Kamilla Muminova, Alena Potapova, Zulfiya Khodzhaeva, Alexey Pyregov, Andrey Elchaninov, Timur Fatkhudinov, Gennady Sukhikh

**Affiliations:** 1 National Medical Research Center for Obstetrics, Gynecology and Perinatology Named after Academician V.I. Kulakov of Ministry of Healthcare of Russian Federation, 117198 Moscow, Russia; a.s.poltavets@gmail.com (A.P.); k_muminova@oparina4.ru (K.M.); doc.potapovaAA@yandex.ru (A.P.); zkhodjaeva@mail.ru (Z.K.); pyregov@mail.ru (A.P.); elchandrey@yandex.ru (A.E.); g_sukhikh@oparina4.ru (G.S.); 2 Histology Department, Peoples’ Friendship University of Russia (RUDN University), 117198 Moscow, Russia; tfat@yandex.ru; 3 Scientific Research Institute of Human Morphology, 117418 Moscow, Russia; mary.krutikova@gmail.com (M.N.); midiberkonst@gmail.com (K.M.); mikhalevalm@yandex.ru (L.M.); 4 Pirogov Russian National Research Medical University (RNRMU), 117997 Moscow, Russia

**Keywords:** macrophages, preeclampsia, decidua, estrogen receptors, polarization, inflammation

## Abstract

Preeclampsia is a gestation-associated hypertensive syndrome that threatens the life and health of the mother and the child. The condition is presumably caused by systemic failure with a strong involvement of innate immunity. In particular, it has been associated with flexible phenotypes of macrophages, which depend on the molecules circulating in the blood and tissue fluid, such as cytokines and hormones. This study aimed at a comparative evaluation of pro-inflammatory (TNFα) and anti-inflammatory (CD206, MMP9, HGF) markers, as well as the levels of estrogen receptor α, expressed by decidual macrophages in normal pregnancy and in patients with early- and late-onset preeclampsia. The tissue samples of decidua basalis were examined by immunohistochemistry and Western blotting. Isolation of decidual macrophages and their characterization were performed using cultural methods, flow cytometry and real-time PCR. Over 50% of the isolated decidual macrophages were positive for the pan-macrophage marker CD68. In the early-onset preeclampsia group, the levels of estrogen receptor α in decidua were significantly decreased. Furthermore, significantly decreased levels of HGF and CD206 were observed in both preeclampsia groups compared with the control group. The observed downregulation of estrogen receptor α, HGF and CD206 may contribute to the balance of pro- and anti-inflammatory macrophages and thereby to pathogenesis of preeclampsia.

## 1. Introduction

The concept of macrophage phenotype plasticity, laid down at the beginning of the 20th century, was able to successfully explain the diversity of macrophage behavior, i.e., why macrophages may either behave as pro-inflammatory cells, or help to resolve inflammation and support tissue regeneration. Over the last decade, abnormal regulation of macrophage plasticity has been increasingly considered causative for a number of pathological conditions. Preeclampsia (PE), one of the so-called “great obstetrical syndromes” [1], is a gestation-associated multisystem disorder which affects from 5% to 7% of all pregnant women and leads to over 70,000 maternal and 500,000 fetal deaths per year around the world [2]. PE is characterized by high maternal blood pressure combined with proteinuria and/or other manifestations. One of the hypotheses on PE etiology is a shift in the balance of pro- and anti-inflammatory macrophages within decidua basalis [3].

Decidua basalis, a part of endometrium interacting with the trophoblast and modified upon the onset of pregnancy, is also known as the maternal part of the placenta. The main cellular components of decidua basalis are macrophages and NK cells promoting immune tolerance of the maternal organism to the fetus and fetal part of the placenta. Abnormal functioning of decidual macrophages may have grave consequences for the pregnancy. A shift in relative numbers of pro- and anti-inflammatory macrophages towards the former may adversely affect the fetal–maternal interface. Abnormal alterations in the local immune responses during trophoblast invasion are fraught with incorrect remodeling of the spiral arteries and impaired transplacental blood flow [4]. An abnormal balance of pro- and anti-inflammatory macrophages in the decidua may result from an abnormal balance of regulatory factors circulating with maternal blood. These factors include cytokines, reactive oxygen species and oxidative stress reporter molecules, elevated levels of which are frequently observed in PE [5,6,7], as well as a decrease in the level of certain hormones, notably estrogens [8,9,10]. Estrogens represent a group of female steroid hormones, exemplified by estrone, estradiol and estriol [11], which play crucial role in pregnancy maintenance. The effects of estrogens are mediated by “classical” receptors estrogen receptor α (ERα) and ERβ, membrane-bound G-protein-coupled estrogen receptor-1 (also known as GPR30), and an ERα variant ERα36 [12]. ERα and ERβ receptors bind estrogens, translocate to the nucleus and bind genomic DNA at estrogen-responsive elements, thus acting as transcription factors [13]. 

A number of studies demonstrated decreased estrogen levels in PE [9,10,14,15]. At the same time, estrogens are implicated in the innate immune signaling in dendritic cells and macrophages as a positive regulator of the anti-inflammatory (M2) polarization. In the work of Dou and colleagues, it was shown that M2 polarization is impaired and M1 polarization is enhanced in estrogen-deficient mice [16]. Sul et al. revealed the estrogen-dependent enhancement of the anti-inflammatory macrophage polarization in adipose tissue [17]. Campbell et al. showed that estrogen and ER-selective agonists promote the anti-inflammatory macrophage activation [18]. 

Macrophages express both ERα and ERβ, but mainly the former type of estrogen receptors [19]. Both ERα and ERβ, as well as the G-protein-coupled estrogen receptor, expressed in endothelial cells and smooth muscle cells of the uterine artery, mediate the vasodilatory effects of estrogens during pregnancy [20]. However, the expression of ERα in human decidua, especially in PE, remains understudied despite the fact that decidua is increasingly becoming a research focus because of its implication in PE. In rats, decidua expresses both ERα and ERβ genes, with the strongest expression observed in the antimesometrial region (where the implantation occurs) [11]. To date, very little attention has been paid to the role of the expression of the estrogen receptor ERα by decidual macrophages in PE. The aim of this study was to investigate the expression of ERα and M2 markers of decidual macrophages during normal pregnancy and in early- and late-onset PE (ePE and lPE, respectively), defined as manifesting before and after 20 weeks of gestation, respectively.

## 2. Materials and Methods

### 2.1. Sample Collection

The Committee on Biomedical Ethics at the Research Center for Obstetrics, Gynecology and Perinatology, Ministry of Healthcare of the Russian Federation, approved the study that was performed according to Good Clinical Practice guidelines and the Declaration of Helsinki (the Ethics Committee Approval Protocol No. 12 of 17 November 2016). Each participant provided informed consent for the study. Placental samples from healthy pregnancies and PE were collected after elective caesarean section. Fragments of decidua (2 × 2 cm^2^) with adjacent fetal material were collected in transport medium (DMEM/F12 with 500 units/mL of penicillin and 500 µg/mL of streptomycin). For immunohistochemical analysis, the fragments of decidua with associated chorionic villi were fixed in 10% formalin. For Western blot analysis, the samples of decidua were snap-frozen in liquid nitrogen.

### 2.2. Isolation of Decidual Macrophages 

The fragments of decidua were cut and scraped. The samples were washed in Hanks’ buffered solution, then incubated in 5 mL of digestion solution containing DNase I (50 mg/mL), collagenase I, II (300 U/mL) in RPMI 1640 culture medium. In total, three rounds of digestion were performed, for 20 min each at 37 °C under stirring, as described elsewhere [21]. After each round of digestion, the supernatant with released cells was collected in a 50 mL tube containing 5 mL of complete growth medium (RPMI 1640 with 10% FBS). The sample was centrifuged at 420× *g* for 11 min at +4 °C. The cells were washed twice with 5 and 10 mL of washing buffer (DMEM/F12 with 2% FBS) and filtered through a 100 µm cell strainer. Mononuclear cells were then separated by Lympholyte-H (Cedarlane, Burlington, ON, Canada) gradient centrifugation and washed with 10 mL of Rinsing Solution (Miltenyi Biotec, Bergisch Gladbach, Germany). The resulting cell suspension was used in further experiments.

### 2.3. Flow Cytometry Analysis 

**Figure 1:**
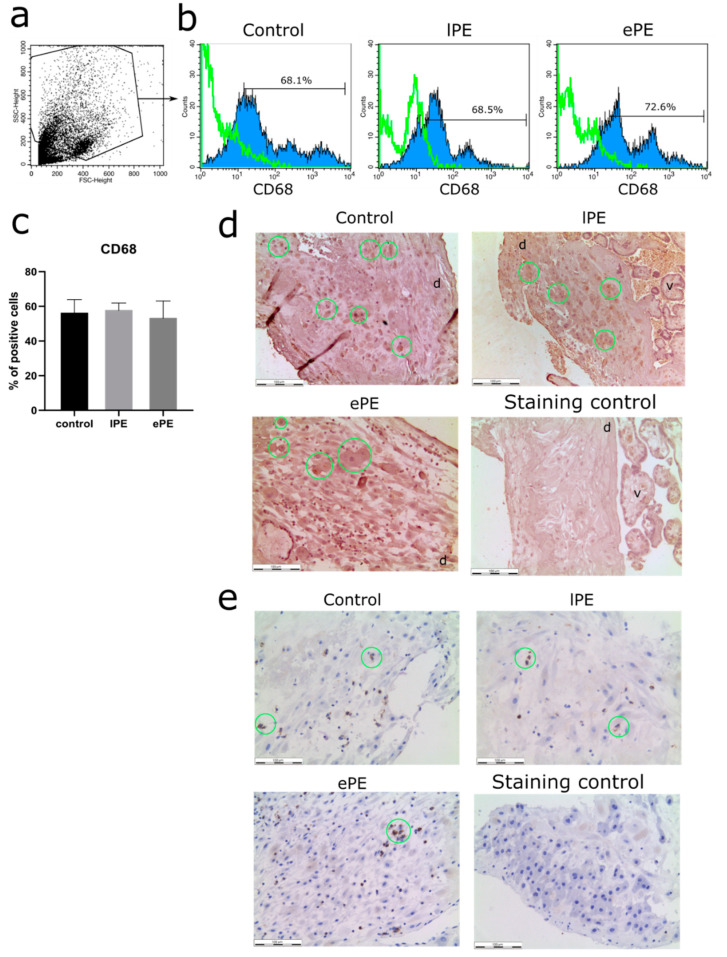
Characterization of the primary cultures of decidual macrophages (**a**–**c**). A representative forward and side scattering dot-plot (**a**). Flow cytometry analysis (**b**), representative histograms are provided for the control, late-onset preeclampsia (lPE) and early-onset preeclampsia (ePE) groups. Green curves represent staining control. The percentages of CD68-positive cells isolated from placenta samples collected from each group are indicated according to flow cytometry analysis (**c**). Immunohistochemical analysis (**d**,**e**), representative photos of decidual membrane stained with hematoxylin and anti-CD68 (**d**) or anti-CD56 (**e**) antibodies (DAB, brown, indicated by green circles), d: decidua, v: villi. Magnification, ×200; bars, 100 µm.

Flow cytometry analysis was performed on a FACScan flow cytometer with CellQuest software (Becton Dickinson, Franklin Lakes, NJ, USA) using monoclonal antibodies to CD68 (130-114-460, clone: REA886, Miltenyi Biotec, Bergisch Gladbach, Germany). The cells (10^5^ per sample) were permeabilized and fixed using the Inside Stain kit (Miltenyi Biotec, Bergisch Gladbach, Germany) according to the manufacturer’s protocols. The cells were gated on the basis of their forward and side scatter properties (Figure 1a) with the region of interest, including main cell population and excluding debris. 

### 2.4. RNA Extraction, Reverse Transcription Reaction, RT-PCR and the Network Analysis

For total RNA extraction from isolated cells, the Extract RNA Reagent (Evrogen, Moscow, Russia) was used according to the manufacturer’s protocol. For reverse transcription, 0.5 μg of total RNA were added per reaction using the MMLV-RT kit (Evrogen, Russia) at 37 °C for 1 h. Real-time PCR was carried out in a 20 μL volume containing 50 ng of cDNA, 0.3 µM oligonucleotide primers and 4 μL of qPCRmix-HS SYBR 5X (Evrogen, Russia), with each reaction set in triplicate. Beta-2 microglobulin (B2M) was applied as a reference gene for estimation of the relative expression values. Primer sequences were as follows: B2M_For 5′-GACTTTGTCACAGCCCAAGAT-3′, B2M_Rev 5′-GTGGAGCAACCTGCTCAGA-3′ (product size 255 bp); ESR1a_For 5′-ATGAAAGGTGGGATACGAAAAGACC-3′, ESR1a_Rev 5′-TGCCAGGTTGGTCAGTAAGCC-3′ (product size 300 bp). Real-time PCR was performed in a DTprime (DT-96) real-time detection thermal cycler (DNA-Technology LLC, Moscow, Russia) using the following program: initial denaturation at 95 °C for 5 min, followed by 30 cycles of denaturation at 94 °C for 15 s, annealing at 62 °C for 10 s and elongation at 72 °C for 20 s. Relative expression levels were determined by the ΔCt method in Q-gene software [22].

### 2.5. Western Blot Analysis 

Pilot cryosections (5 μm) of snap-frozen tissue samples were prepared using a cryotome and stained with hematoxylin to verify the purity of dissected decidual membranes (Figure S1). After that, 5 slices of the material of 50 μm each were prepared and lysed in 100 μL of Protein Solubilization Buffer (PSB, Bio-Rad Laboratories, Inc., Hercules, CA, USA) with the addition of Complete Protease Inhibitor Cocktail (Roche, Basel, Switzerland). Homogenized samples were centrifuged at 14,000× *g* for 30 min. The supernatant was collected, mixed with 2X loading buffer and the sample was incubated at 95 °C for 1 min. The proteins were separated by 10–12.5% sodium dodecyl sulfate polyacrylamide gel electrophoresis (SDS-PAGE) and transferred from the gel to PVDF membranes by a semi-wet approach using the Trans-Blot® Turbo™ RTA Mini LF PVDF Transfer Kit (Bio-Rad Laboratories, Inc., Hercules, CA, USA). A representative image of full-size membrane stained with Ponceau S is available in the Supplementary Material (Figure S2). The membranes were blocked with 5% milk in Tris-buffered saline containing 0.1% Tween for 1 h at room temperature, then incubated overnight with primary antibodies to ERα (ab3575, 1:1000, Abcam, Cambridge, UK), CD206 (ab64693, 1:1000, Abcam), TNFα (ab6671, 1:1000, Abcam), matrix metalloproteinase-9 (MMP9) (ab38898, 1:5000, Abcam), hepatocyte growth factor (HGF) (ab83760, 1:1000, Abcam) and GAPDH (sc-25778, 1:1000, Santa Cruz) overnight at 4 °C with gentle shaking. After that, the membranes were stained with horseradish peroxidase (HRP)-conjugated secondary antibodies (Bio-Rad Laboratories, Inc.) for 1 h at room temperature. The target proteins were visualized by a Novex ECL Kit (Invitrogen™ Thermo Fisher Scientific, Waltham, MA, USA) in the LI-COR visualization system (LI-COR, Lincoln, NE, USA). Image Studio™ Acquisition Software (LI-COR, USA) with GAPDH used as a reference protein was used to measure optical density of the bands. Labeling of Western blot images was conducted in Inkscape software with minimal processing. 

### 2.6. Immunohistochemistry

The formalin-fixed placenta samples underwent routine histological processing. For immunohistochemical analysis, paraffin sections (5 μm) were stained with primary antibodies to ERα (ab3575, 1:100, Abcam), CD68 (ab125212, 1:100, Abcam), CD206 (ab64693, 1:100, Abcam) manually. The immunoperoxidase-stained sections were counterstained with Mayer’s hematoxylin. Paraffin sections were stained with primary to CD56 (NCL-CD56-504, clone CD564, Leica Biosystems, Wetzlar, Germany) in a Leica BOND-MAX Automated Immunostainer with further incubation with immunoperoxidase-conjugated antibodies and counterstaining with hematoxylin included in the CD56 staining kit. For each section, 10 images were obtained on a Leica DM2500 system. 

### 2.7. Statistical Analysis

For data presentation, the mean ± standard error mean (SEM) and the median with interquartile range values were calculated. The Shapiro–Wilk test was used to evaluate normality of the distributions. One-way analysis of variance (ANOVA) followed by Dunnett’s post hoc test was used to identify differences among multiple groups with normal distributions. Kruskal–Wallis non-parametric ANOVA followed by the post hoc Dunn test was used to calculate statistical differences for non-normal distributions. The data were analyzed in Prism 7.0 software (GraphPad, San Diego, CA, USA). All comparisons were made at a significance level of 0.05.

## 3. Results

Based on the anamnestic and clinical data, the participants (*n* = 30) were assigned into three groups, corresponding to normal pregnancy, who, according to clinical data, underwent caesarean section (control group) and early- and late-onset PE, with ten individuals in each group. Clinical and demographic characteristics of the participants are given in Table 1. Significant differences in gestational age at delivery, blood pressure, urinary protein levels and newborn body weight were observed between the groups. The records of intrauterine growth restriction, hypertension prior to the pregnancy and miscarriage were confined to PE groups.

### 3.1. Decidual Macrophages in Primary Culture

By following the isolation protocol, we obtained cultures of decidual macrophages which were subsequently analyzed by flow cytometry. Immunostaining for the common macrophage marker CD68 showed that over 50% of the isolated cells were positive, and this index did not differ significantly between the groups (Figure 1b,c). Applying the antibody against the same antigen for immunohistochemistry allowed us to visualize macrophages (green circles in Figure 1d) in the decidua (designated by “d” in Figure 1d). We also observed a minor portion of NK cells in the decidua (CD56 positive, green circles in Figure 1e).

### 3.2. Downregulation of ERα and HGF Levels in Decidual Macrophages

**Figure 2:**
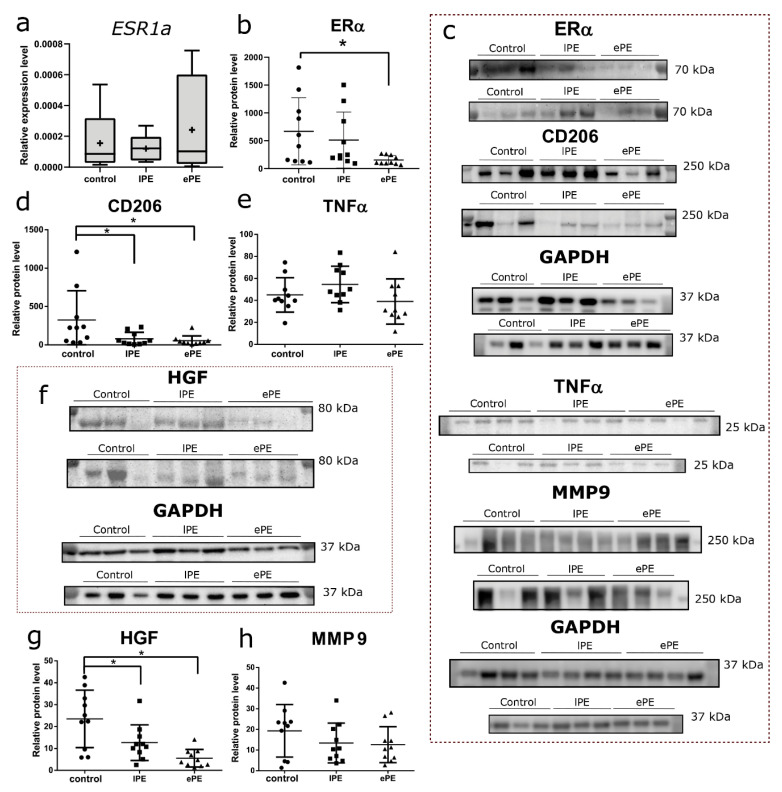
PCR and Western blot analysis of gene and protein expression by decidual macrophages for the control, late-onset preeclampsia (lPE) and early-onset preeclampsia (ePE) groups. Relative expression levels of *ESR1a* gene in the isolated decidual macrophages (**a**). Relative protein levels of ERα (**b**), CD206 (**d**), TNFα (**e**), HGF (**g**) and MMP9 (**h**) in decidua basalis, normalized by GAPDH level. Representative Western blotting membranes stained with antibodies to ERα, CD206, TNFα, GAPDH and MMP9 (**c**), HGF and GAPDH (**f**). * *p* < 0.05 compared to the control group according to ANOVA with Dunnett’s post hoc test.

In the next stage of the study, we performed comparative analysis of expression for the *ESR1a* gene encoding estrogen receptor alpha (ERα) in macrophages isolated from decidua. At the mRNA level, *ESR1a* was expressed similarly in all groups (Figure 2a). Western blot analysis, however, revealed reduced levels of ERα in decidua samples of the ePE group compared with the control (*p* = 0.04; Figure 2b,c). We also analyzed the levels of CD206, a mannose receptor and marker of the anti-inflammatory phenotype of macrophages. Significantly decreased levels of CD206 were observed in both PE groups compared with the control (*p* = 0.04 for lPE and *p* =0.02 for ePE; Figure 2c,d). At the same time, the levels of pro-inflammatory cytokine TNFα in all groups were similar (Figure 2c,e), as well as the levels of MMP9, which is considered as a marker of the anti-inflammatory polarization of macrophages (Figure 2c,h). The content of HGF protein was significantly decreased in both PE groups compared with the control (*p* = 0.02 for lPE and *p* = 0.0003 for ePE; Figure 2f,g). 

### 3.3. ERα and CD206 Immunostaining in Placental Tissue Sections

**Figure 3:**
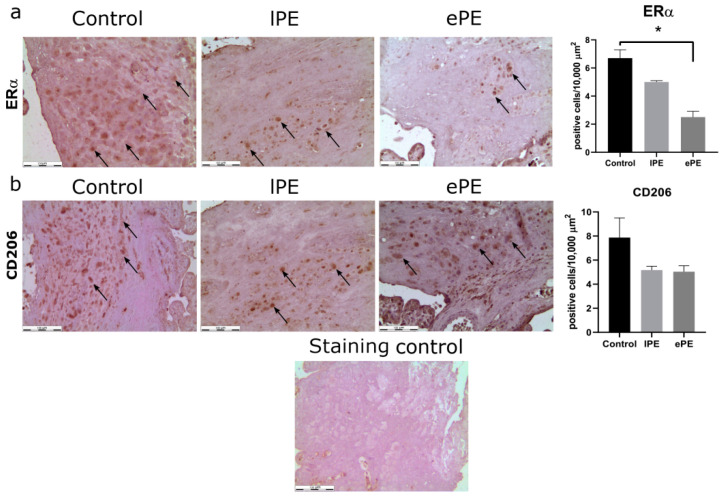
Immunohistochemical analysis of ERα- and CD206-positive cells in decidua basalis of lPE, ePE and the control placenta samples. The percentages of ERα- and CD206-positive cells per 10,000 μm^2^ are indicated. Representative photos of sections stained with hematoxylin and anti-ERα (**a**) and anti-CD206 (**b**) antibodies (DAB, brown, indicated by black arrows). Magnification, ×200; bars, 100 µm. * *p* < 0.05 vs. control group according to Kruskal–Wallis non-parametric ANOVA with post hoc Dunn test.

Immunohistochemistry was used as an additional method to verify changes in the level of ERα production by decidual macrophages. Interestingly, the number of positively stained cells was significantly lower in the ePE group compared to the control group (*p* = 0.01) (Figure 3a). Additionally, we performed immunohistochemistry for the CD206 marker (Figure 3b). Despite the decrease in its production in both groups with PE, the differences were not statistically significant.

## 4. Discussion

By now, PE remains the leading cause of maternal and fetal morbidity and mortality [23]. PE has been comprehensively studied and characterized in terms of markers found in different fractions of the blood, as well as in exosomes and urine [24,25]. However, more attention should be paid to molecular-level events in the placenta and adjacent maternal organs as the site of PE pathogenesis. Very little was found in the literature on the question of the role of the decidual membrane of the placenta in PE. Macrophages constitute a prominent cellular component of the decidua, along with NK cells [26]. In this study, we focused on decidual macrophages in normal pregnancy compared with the early- and late-onset PE. The results indicate significantly decreased levels of ERα, HGF and CD206 in decidual macrophages for both types of PE, and no significant alterations in the levels of MMP9 and TNFα compared with normal pregnancy.

Over 50% of the isolated cells in all studied groups were positive for the pan-macrophage glycoprotein CD68; this was confirmed by flow cytometry and immunohistochemical staining. The apparent inconsistency of this finding with the previously reported evidence on the increased numbers of decidual CD68-positive cells in PE [27,28] can be explained by the use of different techniques and criteria for patient group formation. Al-khafaji and colleagues, for example, did not report whether the PE group included early- or late-onset PE or mixed-term PE [28]. Milosevic-Stevanovic et al. compared decidua of the control patients with late-onset PE [27] and both research groups used only an immunohistochemistry approach without flow cytometry analysis, which was used in our study. 

The role of decidual macrophages in PE is currently being focused on more closely. The anti-inflammatory phenotype of decidual macrophages is a hallmark of normal pregnancy [29,30,31], whereas PE has been associated with a shift in the balance of pro- and anti-inflammatory macrophages, which entails an exacerbation of the local immune response in the placenta with the release of inflammatory cytokines [29,32,33]. A study by Schonkeren and colleagues revealed a PE-specific decrease in the number of decidual cells positive for the anti-inflammatory phenotype marker CD163 [34]. Wheeler et al. demonstrated that, in PE, decidual macrophages predominantly express increased levels of pro-inflammatory markers (CXCL10 and IL12) and decreased levels of anti-inflammatory markers (CD206 and CCL17) as measured by RT-PCR [35]. In this study, we demonstrated a decrease in the level of CD206 production by decidual macrophages in the early- and late-onset PE groups by Western blotting. 

Furthermore, we observed no significant changes in the production of TNFα and MMP9, along with significantly decreased HGF levels in both PE groups. HGF, though known specifically as a hepatocyte growth factor, acts as a pleiotropic cytokine; it supports morphogenesis, regeneration and cell survival in various tissues [36]. HGF is essential for human placenta organogenesis and especially for extravillious trophoblast invasion [37]. The observed decrease in HGF levels is consistent with the observations of Furugori et al. concerning the downregulation of HGF mRNA and protein content in preeclamptic placentas [38]. Interestingly, enhanced HGF production is characteristic of the M2-induced polarization of peritoneal macrophages [39]. This finding, while preliminary, suggests that the opposite effect observed in preeclamptic placentas can be associated with a local decline in the anti-inflammatory polarization of macrophages. 

Macrophage polarization is known to be strongly influenced by circulating levels of sex hormones, notably estrogens [40]. In pregnancy, estrogens are responsible for enhancing uterine angiogenesis at the implantation site, as well as for the maintenance of uteroplacental blood flow and tissue remodeling of the mammary glands [41]. Estrogens act on endothelial and endometrial cells and have been shown to induce the expression of pro-angiogenic molecules such as VEGF and TGF-β [42], the latter being one of the key factors responsible for the anti-inflammatory polarization of macrophages [40,43]. Estrogens can also induce the anti-inflammatory phenotypes directly, which has been confirmed by several studies [40,44,45]. It is important that estrogen levels in PE are decreased [9,10,14,15,46]. Evidence that estrogens induce expression of their own cognate receptors has been presented by other authors [47,48,49]. Here, we report a significant decrease in the ERα levels expressed by decidual macrophages in ePE. These results are difficult to compare with published data as, for the decidua, a decrease in the production of the ERα in ePE is demonstrated for the first time. 

Most of the published studies on the expression of estrogen receptors in the placenta deal with its fetal part. This is explained by the fact that the fetal part is much larger in size than the maternal part and is technically easier to work with. However, the chorionic villi have a less homogeneous composition than the maternal decidua basalis and are represented mainly by syncytiotrophoblast and vascular cells. Data on the content of different types of estrogen receptors in the placenta in PE vary. For example, Yin et al. observed significantly higher ERα levels within the fetal part of the placenta in severe PE compared with the control group [50]. This finding is especially interesting given the reduced placental levels of another estrogen receptor, GPR30, observed by other authors in both the chorionic villi and decidua of PE patients [51]. It has also been reported that the risk of developing severe PE is associated with polymorphisms in the *ESR1* gene [52] which could affect production of the receptor itself. 

Comparative studies on the estrogen receptor expression in different cells of the monocyte–macrophage system are scarce. Monocytes express both ERα and ERβ at low levels [53]. In mature cells of the monocyte–macrophage system, expression levels of ERα and ERβ differ. For example, dendritic cells express predominantly ERα [54]. However, upon migration to the brain, dendritic cells start to express ERβ along with ERα, i.e., relative levels of ER expression may depend on the microenvironment [55].

Considering the pronounced effects of estrogens on macrophage phenotypes (including the profiles of cytokine gene expression), it can be assumed that, in macrophages, estrogen receptors are expressed more strongly than in monocytes (in which the levels of ERα and ERβ are low [53]). Indeed, the differentiation of monocytes into macrophages is accompanied by an increase in ER levels, as confirmed by in vitro studies on human CD14+ monocyte-derived macrophages and their activation with IFN-γ [53]. Given the hypoestrogenism observed in PE, it would be interesting to compare expression levels of ERα and ERβ in monocytes before and after menopause. In the hypoestrogenic state of the body associated with menopause, monocytes predominantly express ERα, and at higher levels than in premenopausal women with normal blood estrogen levels [53].

Thus, we can conclude that the reduced expression of estrogen receptors by decidual macrophages in ePE, as opposed to the steady increase in ER expression normally observed during the differentiation of monocytes into macrophages, indicates a dysfunction of the decidual macrophage population in PE patients. The observed decrease in ER expression levels correlates with the data on HGF production. Enhanced production of HGF by peritoneal macrophages in endometriosis (which is often associated with the hyperestrogenism-induced M2 shift in macrophage polarization) was demonstrated in several studies [56,57]. Experiments by Khan et al. showed that peritoneal macrophages isolated from endometriosis patients produced significantly higher levels of HGF after exposure to estradiol than without it [58]. We therefore suggest that the observed downregulation of ER expression and HGF production by decidual macrophages results from the decreased estrogen levels characteristic of PE. The ultimate effect is imbalance in pro- and anti-inflammatory macrophage phenotypes, which could be considered as one of the key stages in PE pathogenesis. 

Certain shortcomings of this study should be mentioned, notably, the technique used for macrophage isolation and the lack of gestational age-matched controls for the ePE group. Concerning the latter, we considered it improper to use the age-matched control for ePE due to the obvious impossibility of excluding other factors associated with preterm delivery (placental abruption, uterine overdistention, cervical incompetence, hormonal imbalance, infections, etc. [59]). The yields of resident macrophages from placental tissues can be incomplete due to the loss of monocytic markers (CD14/CD16/CD11b) by the cells in the course of infiltration.

## 5. Conclusions

The results support the idea that the PE-associated decrease in ERα expression (observed in our study) may impair anti-inflammatory polarization of decidual macrophages and thus contribute to PE pathogenesis. The study raises important questions on the expression of other types of estrogen receptors in the decidua and underscores the necessity of the comprehensive phenotypic characterization of decidual macrophages. 

## Figures and Tables

**Table 1:** Clinical characteristics of the participants.

Characteristics	Control	lPE	ePE
Number	10	10	10
Maternal age, years	33.9 ± 3.5	34.6 ± 5.2	33.7 ± 6.3
Gestational age at the moment of c/s, weeks	38.6 ± 0.7	36.8 ± 1.4 *	30.2 ± 3.4 *
Systolic blood pressure, mm Hg	115.1 ± 5.2	149.5 ± 26.1 *	158.1 ± 16.4 *
Diastolic blood pressure, mm Hg	73.6 ± 3.0	94.8 ± 13.3 *	99.6 ± 11.5 *
Proteinuria, g/L	not detected	1.567 ± 2.2	2.516 ± 2.2
Newborn mass, g	3551.5 ± 469.02	2261.4 ± 463.7 *	1325.1 ± 56.2 *
Newborn gender (Male/Female)	4/6	3/7	4/6
Intrauterine growth restriction, number of cases	0	5	3
Diabetes cases	0	0	0
Hypertension prior to the pregnancy, number of cases	0	2	2
Previous pregnancies, min–max and most frequent value in group	0–41 and 2	0–30	0–20
Miscarriage, number of cases	0	2	1

* *p* < 0.01 vs. control group according to Kruskal–Wallis non-parametric ANOVA with post hoc Dunn test. c/s—caesarean section.

## Data Availability

Data is contained within the article or supplementary material.

## Supplementary Materials

The following are available online https://www.mdpi.com/2227-9059/9/2/191/s1. Figure S1, Representative slices of snap-frozen placenta samples from the late-onset (lPE) and early-onset (ePE) patients and women with physiological pregnancy (control), for verification of decidual membrane; Figure S2, Full-size membrane after blotting of polyacrylamide gel with placenta samples from the control group, late-onset PE (lPE) and early-onset PE (ePE).
