# Supplementary material for: Expression of Estrogen Receptor α by Decidual Macrophages in Preeclampsia
Source: Biomedicines. 2021 Feb 14;9(2):191. doi: 10.3390/biomedicines9020191 (PMC7917975; doi:10.3390/biomedicines9020191)
Supplement: Supplementary file 1 [file biomedicines-09-00191-s001.pdf]

Supplementary data

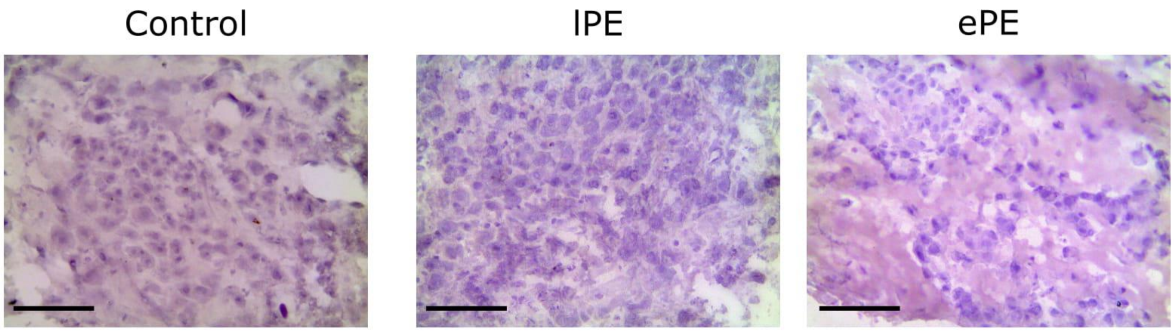

Figure S1 Representative slices of snap-frozen placenta samples from the late-onset (lPE) and early-onset (ePE) patients and women with physiological pregnancy (control), for verification of decidual membrane. Slices stained with hematoxylin. Magnification, ×200; bars, 100 μm.

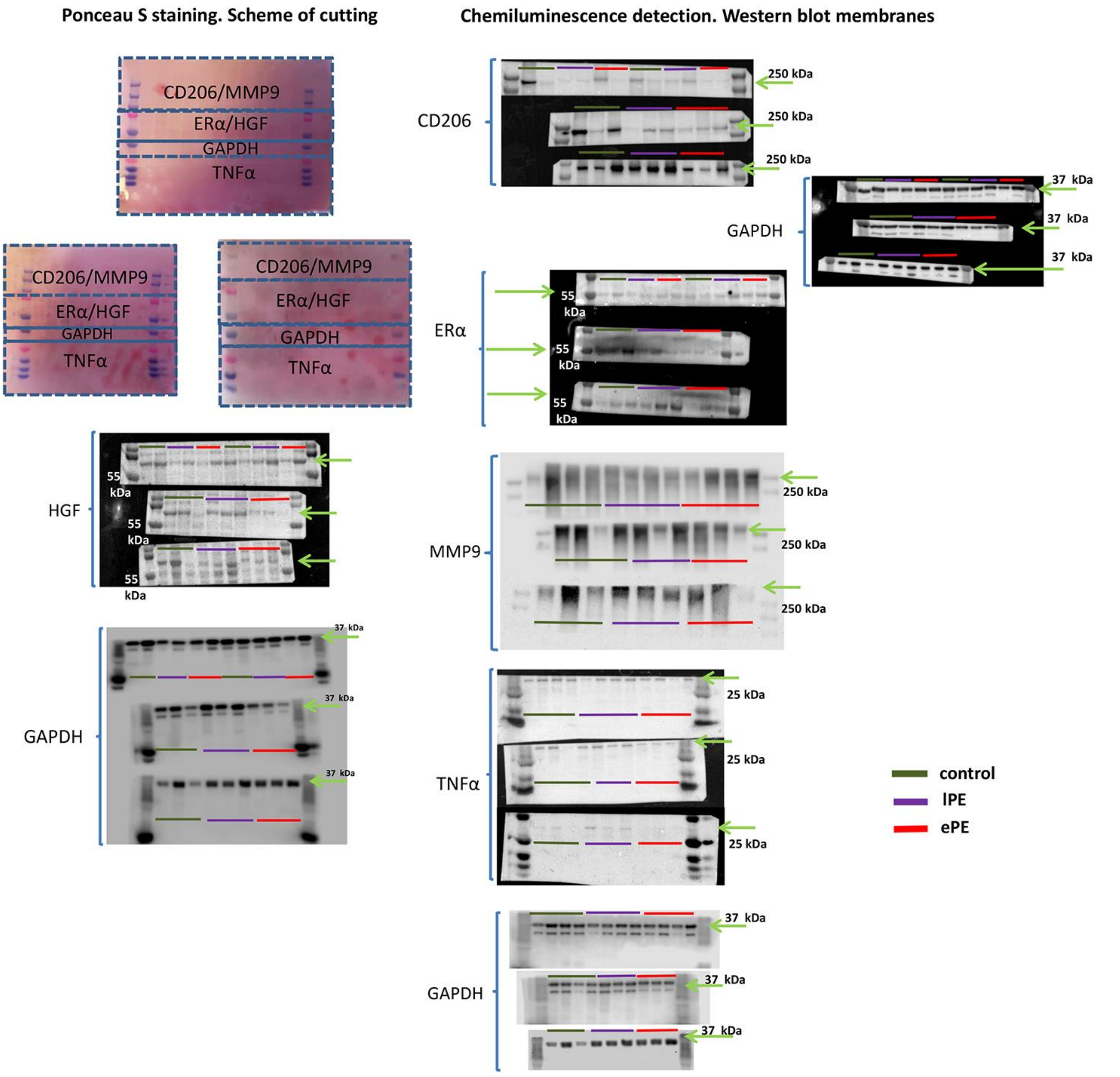

Figure S2 Full-size membrane after blotting of polyacrylamide gel with placenta samples from the control group, late-onset PE (lPE) and early-onset PE (ePE). Schemes of membrane Ponceau S staining and cutting are shown. After visualization of the proteins with Ponceau S membranes were cut as indicated with a dotted line and stained with the mentioned antibody. The analyzed band is marked with a green arrow, position of samples from the concrete group is marked with color line.
